# Clinical presentation and therapeutic management of venous thrombosis in young children: a retrospective analysis

**DOI:** 10.1186/s12959-018-0182-4

**Published:** 2018-11-01

**Authors:** Anthony Chan, Anthonie W. A. Lensing, Dagmar Kubitza, Grahaem Brown, Dolores Elorza, Marta Ybarra, Jacqueline Halton, Sebastian Grunt, Gili Kenet, Damien Bonnet, Amparo Santamaria, Paola Saracco, Tina Biss, Francesco Climent, Philip Connor, Joseph Palumbo, Kirstin Thelen, William T. Smith, Amy Mason, Ivet Adalbo, Scott D. Berkowitz, Eva Hurst, Jeroen van Kesteren, Guy Young, Paul Monagle

**Affiliations:** 1 McMaster Children’s Hospital, Hamilton, Canada; 2 Bayer AG, Wuppertal, Germany; 3 Bayer U.S., LLC, Whippany, USA; 4 Competitive Drug Development International Ltd. (CDDI), London, UK; 5 Hospital Universitario La Paz, Madrid, Spain; 6 Children’s Hospital of Eastern Ontario (CHEO), University of Ottawa, Ottawa, Canada; 7 Division of Neuropaediatrics, Development and Rehabilitation, University Children’s Hospital, University of Bern, Bern, Switzerland; 8 Sheba medical center, Ramat Gan, Israel; 9 Hôpital Necker- Enfants Malades, Paris, France; 10 Hospital Vall d’Hebron, Barcelona, Spain; 11 University Hospital, Città della Salute e della Scienza di Torino, Ospedale Infantile Regina Margherita, Torino, Italy; 12 The Newcastle upon Tyne Hospitals NHS Foundation Trust, Newcastle upon Tyne, UK; 13 Hospital Universitario La Paz, Madrid, Spain; 14 The Noah’s Ark Children’s Hospital for Wales, University Hospital of Wales, Cardiff, UK; 15 Cincinnati Children’s Hospital Medical Center, University of Cincinnati College of Medicine, Cincinnati, USA; 16 Hemostasis and Thrombosis Center (HTC), Children’s Hospital Los Angeles, Los Angeles, USA; 17 Department of Haematology, Royal Children’s Hospital, Department of Paediatrics, University of Melbourne, Murdoch Children’s Research Institute, Melbourne, Australia; 18 Research and Development, Thrombosis and Hematology, Building 402, room 304, Aprather Weg 18a, 42113 Wuppertal, Germany

**Keywords:** Venous thromboembolism (VTE), Pediatric trial, Rivaroxaban, Direct oral anticoagulant (DOAC/NOAC), Anticoagulation, Registry

## Abstract

**Background:**

Venous thromboembolism (VTE) in young children is not well documented.

**Methods:**

Clinicians from 12 institutions retrospectively evaluated the presentation, therapeutic management, and outcome of VTE in children younger than 2 years seen in 2011–2016. Feasibility of recruiting these children in EINSTEIN-Jr. phase III, a randomized trial evaluating rivaroxaban versus standard anticoagulation for VTE, was assessed.

**Results:**

We identified 346 children with VTE, of whom 227 (65.6%) had central venous catheter-related thrombosis (CVC-VTE), 119 (34.4%) had non-CVC-VTE, and 156 (45.1%) were younger than 1 month. Of the 309 children who received anticoagulant therapy, 86 (27.8%) had a short duration of therapy (i.e. < 6 weeks for CVC-VTE and < 3 months for non-CVC-VTE) and 17 (5.5%) had recurrent VTE during anticoagulation (*n* = 8, 2.6%) or shortly after its discontinuation (*n* = 9, 2.9%). A total of 37 (10.7%) children did not receive anticoagulant therapy and 4 (10.5%) had recurrent VTE.

The average number of children aged < 0.5 years and 0.5–2 years who would have been considered for enrolment in EINSTEIN-Jr is approximately 1.0 and 0.9 per year per site, respectively.

**Conclusions:**

Young children with VTE most commonly have CVC-VTE and approximately one-tenth and one-fourth received no or only short durations of anticoagulant therapy, respectively. Recurrent VTE rates without anticoagulation, during anticoagulation or shortly after its discontinuation seem comparable to those observed in adults. Short and flexible treatment durations could potentially increase recruitment in EINSTEIN-Jr. phase III.

## Background

Pediatric anticoagulant guidelines for venous thromboembolism (VTE) are mainly based on extrapolation from trials in adults, with low levels of evidence from the pediatric population. However, anecdotal evidence in neonates resulted in suggested (Grade 2 C recommendations) treatment durations of between 6 weeks and 3 months for both central venous catheter-related VTE (CVC-VTE) and other VTE (e.g. renal vein thrombosis or cerebral sinus venous thrombosis) [1]. In clinical practice, shorter durations of treatment (i.e. < 3 months) are frequently used in children beyond the neonatal age group, presumably related to the difficulties in delivering anticoagulation using currently available drugs.

Performing clinical studies in young children with VTE is challenging for a variety of reasons. First, VTE is a relatively rare condition in children with reported incidences being approximately 100 times lower than in adults, requiring large and expensive collaborative efforts to recruit even a low number of children [2–8]. Second, young children with VTE represent a sick population since their VTE is often hospital acquired, largely due to more aggressive treatments of serious and life-threatening comorbidities [9]. Third, international restrictions on blood volumes that can be drawn for purposes other than routine medical care, limit participation of those with a low bodyweight [10]. Finally, parental consent is often difficult to obtain in young children with acute serious conditions in combination with a short decision window.

The EINSTEIN-Jr phase III study (clinicaltrials.gov NCT02234843) is a randomized study comparing the efficacy and safety of bodyweight-adjusted rivaroxaban with standard anticoagulation for the treatment of acute VTE in children [11]. The study started in 2014 with children aged 12–18 years and, in a staggered approach, continued with those aged 6–12 years, and 2–6 years. Study treatment is given for 3 months with the option to continue treatment in 3 months increments, up to a total duration of 12 months, with a repeat imaging test scheduled at month 3. During the last phase of the EINSTEIN-Jr. study, recruitment will be opened for neonates and infants younger than 2 years.

In this retrospective analysis, we describe the presentation, therapeutic management, and outcomes observed in children younger than 2 years who received care for VTE in recent years. In addition, we assessed their potential eligibility for enrolment into the EINSTEIN-Jr trial.

## Methods

A group of EINSTEIN-Jr. investigators from 12 institutions in North America, Europe and Israel collected data from their institutions for the period 2011–2016 for children younger than 2 years with VTE and described the diagnosis of VTE at the time of presentation, if anticoagulant therapy was instituted, and the type of therapy. In addition, they assessed if children had developed recurrent VTE.

The potential eligibility for the EINSTEIN-Jr study of children with VTE seen in 2016 was done by determining the proportion of children who would have passed the screen of inclusion and exclusion criteria, as proposed in the protocol (Table 1). Then, investigators indicated whether or not they would have considered each child for inclusion into the study. This question was intended to capture additional factors not specified by the eligibility criteria, such as the child being considered too ill, or because the child was discharged to a remote location.

**Table 1:** Inclusion and exclusion criteria of the EINSTEIN Jr. study in children younger than 2 years as evaluated in this feasibility assessment

Eligibility criteria	Rationale
Inclusion criteria for children aged < 0.5 year	
Confirmed VTE and initial treatment with therapeutic dosages of UFH, LMWH or fondaparinux and requirement for anticoagulant therapy for at least 3 months (at least 6 weeks for those with catheter-related VTE)	Target population
Gestational age at birth of at least 37 weeks	Maturation of organs involved in rivaroxaban absorption and clearance depend on the gestational and postnatal age. Rivaroxaban PK variability is expected to be higher in children born preterm compared to term neonates and older children [15–21].
Oral, nasogastric or gastric feeding for at least 10 days	Literature data indicates that gastrointestinal conditions are more stable in children with a gestational age of ≥37 weeks who have been on oral feeding for at least 10 days [15–21]. Rivaroxaban should be taken with food to achieve optimal absorption [22, 23].
Bodyweight ≥2600 g	Above 2600 g representative virtual children could be simulated with the rivaroxaban PBPK model for (term born) neonates
Inclusion criteria for children aged 0.5–2 years	
Confirmed VTE and initial treatment with therapeutic dosages of UFH, LMWH or fondaparinux and requirement for anticoagulant therapy for at least 3 months (at least 6 weeks for those with catheter-related VTE)	Target population
Exclusion criteria	
Active bleeding or bleeding risk contraindicating anticoagulant therapy	Potential risk factor for (increased) bleeding with any anticoagulant
Estimated glomerular filtration rate < 30 mL/min/1.73m^2^ (in children < 1 year, serum creatinine results above 97.5th percentile [24, 25]	Potential risk factor for bleeding with any anticoagulant
Hepatic disease associated with either a coagulopathy leading to a clinically relevant bleeding risk, or ALT > 5× ULN	Potential risk factor for bleeding with any anticoagulant
Platelet count < 50 × 10^9^/L	Potential risk factor for bleeding with any anticoagulant
Sustained uncontrolled hypertension defined as systolic and/or diastolic blood pressure > 95th age percentile [26]	Potential risk factor for bleeding with any anticoagulant
Life expectancy < 3 months	A priori likelihood for the child to not complete the study
Concomitant use of strong inhibitors of both CYP3A4 and P-gp;	Potential for increased rivaroxaban plasma concentrations to a clinically relevant degree
Concomitant use of strong inducers of CYP3A4	Potential for reduced rivaroxaban plasma concentrations
Gastrointestinal disease associated with impaired absorption	Potential for reduced rivaroxaban plasma concentrations
Hypersensitivity or any other contraindication listed in the local labeling for rivaroxaban or comparator treatment	Contraindication for use of the product
Participation in a study with an investigational drug or medical device within 30 days prior to randomization	Regulatory requirement

VTE denotes venous thromboembolism, UFH unfractionated heparin, LMWH low molecular weight heparin, ALT alanine aminotransferase, ULN upper limit of normal, CYP 3A4 cytochrome P450 isoenzyme 3A4, P-gp P-glycoprotein

Data that was collected from the records included year of diagnosis, site of venous thrombosis, gestational age and bodyweight at birth, age at diagnosis, type of anticoagulant treatment (if given), and occurrence of recurrent VTE. For the purposes of this study, short duration treatment was defined a priori as < 6 weeks for those CVC-VTE and < 3 months for those with non-CVC-VTE. In addition, other details available to address the set of eligibility criteria were collected. To better describe factors which may contribute to children being rendered ineligible for participation in EINSTEIN-Jr, the cumulative loss of children for the year 2016 is depicted in a funnel diagram. For the latter analysis, children for whom missing data was reported were assumed to have fulfilled criteria.

The search strategies to identify children with VTE varied between the sites and consisted of review of anticoagulation prescription records from the hospital pharmacy database, the investigator’s referral records, discharge letters, and department’s patient records. At a single site, data was collected for the first 2 months of each year and, therefore, does not represent a complete sample over the time period 2011–2016.

The study was conducted by Competitive Drug Development International Ltd. (CDDI), London, United Kingdom in collaboration with Bayer AG. The protocol was approved by the Institutional Review Board or Ethics Committee of each participating center, when required.

### Data handling

De-identified data was retrieved and entered into a validated electronic data capture system at the investigator site and, once queries had been clarified, downloaded into an Excel workbook. Results were calculated as percentages.

## Results

### Patients

**Fig. 1:**
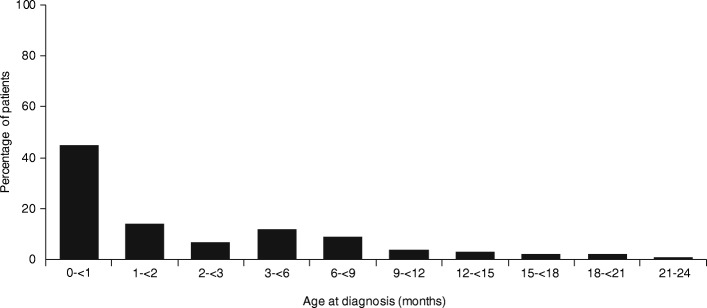
Age of children younger than 2 years diagnosed in 2011–2016 (*N* = 346)

A total of 346 children younger than 2 years were identified of whom 227 (65.6%) had CVC-VTE and 119 (34.4%) had non-CVC-VTE (Table 2). The age distribution of these children is shown in Fig. 1. VTE was most prevalent during the first months of life with 156 (45.1%) being younger than 1 month. Most children were diagnosed with VTE in neonatal or pediatric intensive or high care units (91% of children < 0.5 years and 67% of children aged 0.5–2 years). The most frequently involved anatomical sites in the CVC-VTE group were the extremities, caval vein or lungs (123, 54.2%), jugular or subclavian vein (61, 26.9%), and the heart (24, 10.6%; Table 2). The most frequently involved anatomical sites in the non-CVC-VTE group were the cerebral veins and sinuses (45, 37.8%), and extremities, caval vein or lungs (27, 22.6%; Table 2).

**Table 2:** Type of anticoagulant therapy given for children younger than 2 years diagnosed with VTE

	Total population, *N* = 346	Children aged < 0.5 year, *N* = 271	Children aged 0.5-2 years, *N* = 75
CVC-VTE, *n* (%)	227 (65.6)	175 (64.6)	52 (69.3)
Anticoagulation,^a^ *n* (%)	199 (88)	153 (87)	46 (88)
No anticoagulation, *n* (%)	28 (12)	22 (13)	6 (12)
Non-CVC-VTE, *n* (%)	119 (34.4)	96 (35.4)	23 (30.7)
Anticoagulation,^a^ *n* (%)	110 (92)	88 (92)	22 (96)
No anticoagulation, *n* (%)	9 (8)	8 (8)	1 (4)
CVC-VTE			
Extremity/caval vein thrombosis/pulmonary embolism		89	34
Anticoagulation,^a^ *n* (%)		76 (85)	30 (88)
No anticoagulation, *n* (%)		13 (15)	4 (12)
Cardiac thrombosis		20	4
Anticoagulation,^a^ *n* (%)		18 (90)	4 (100)
No anticoagulation, *n* (%)		2 (10)	0
Renal vein thrombosis		1	0
Anticoagulation,^a^ *n* (%)		1 (100)	–
No anticoagulation, *n* (%)		0	–
Portal vein thrombosis		18	0
Anticoagulation,^a^ *n* (%)		14 (78)	–
No anticoagulation, *n* (%)		4 (22)	–
Jugular/subclavian vein thrombosis		47	14
Anticoagulation,^a^ *n* (%)		44 (94)	12 (86)
No anticoagulation, *n* (%)		3 (6)	2 (14)
Non-CVC-VTE			
Cerebral venous sinus thrombosis		37	8
Anticoagulation,^a^ *n* (%)		34 (92)	8 (100)
No anticoagulation, *n* (%)		3 (8)	0
Extremity/caval vein thrombosis/pulmonary embolism		16	11
Anticoagulation,^a^ *n* (%)		14 (88)	11 (100)
No anticoagulation, *n* (%)		2 (13)	0
Cardiac thrombosis		12	0
Anticoagulation,^a^ *n* (%)		12 (100)	–
No anticoagulation, *n* (%)		0	–
Renal vein thrombosis^b^		14	0
Anticoagulation,^a^ *n* (%)		12 (86)	–
No anticoagulation, *n* (%)		2 (14)	–
Portal vein thrombosis^b^		14	1
Anticoagulation,^a^ *n* (%)		14 (100)	0
No anticoagulation, *n* (%)		0	1 (100)
Jugular/subclavian vein thrombosis		4	3
Anticoagulation,^a^ *n* (%)		3 (75)	3 (100)
No anticoagulation, *n* (%)		1 (25)	0

^a^Unfractionated heparin, low molecular weight heparin, fondaparinux or vitamin K antagonists. ^b^A single patient had renal and portal vein thrombosis and was therefore considered in both groups

### Anticoagulant therapy

A total of 37 (10.7%) of the 346 children did not receive anticoagulant treatment (Table 2). Of the 309 children who had anticoagulant therapy, 282 (91.3%) received low molecular weight heparin, 24 (7.8%) unfractionated heparin, 2 (0.6%) fondaparinux, and 1 (0.3%) received only therapy with vitamin K antagonists, whereas 6 (1.9%) children transitioned from heparins to vitamin K antagonists. Type of anticoagulant and treatment durations were similar for the various manifestations of venous thrombosis and across age groups (Tables 2 and 3).

**Table 3:** Short durations of anticoagulant treatment in relation to presenting VTE and age

	Duration anticoagulation shorter than 6 weeks (CVC-VTE group) or shorter than 3 months (non-CVC-VTE group)	
	Children aged < 0.5 year (*n* = 241)	Children aged 0.5–2 years (*n* = 68)
CVC-VTE, *n* = 199	36/153 (24)	8/46 (17)
Non-CVC-VTE, *n* = 110	35/88 (40)	7/22 (32)

CVC-VTE denotes central venous catheter related venous thromboembolism

### Efficacy outcomes

Of the 309 children who received anticoagulation, 17 (5.5%) had a symptomatic recurrent VTE (Table 4), which occurred during anticoagulation in 8 (2.6%; CVC-VTE group, 6/199 (3.0%); non-CVC-VTE group, 2/110 (1.8%)) and following discontinuation of anticoagulation in 9 (2.9%; CVC-VTE group, 8/199 (4.0%); non-CVC-VTE group, 1/110 (0.9%)). Of the 37 children who were not treated with anticoagulants, 4 (10.5%); had a recurrent VTE (all 4 were in the CVC-VTE group, 4/28 (14.3%); non-CVC-group, 0/9 (0%)). Results were similar across age categories (data not shown).

**Table 4:** Recurrent VTE in relation to presence or absence of anticoagulant therapy

	CVC-VTE	Non-CVC-VTE
	*N* = 227	*N* = 119
During anticoagulation, n/N (%)	6/199 (3.0)	2/110 (1.8)
Following discontinuation of anticoagulants, n/N (%)	8/199 (4.0)	1/110 (0.9)
No anticoagulant group, n/N (%)	4/28 (14.3)	0/9 (0)

### Reasons for ineligibility for EINSTEIN-Jr

**Fig. 2:**
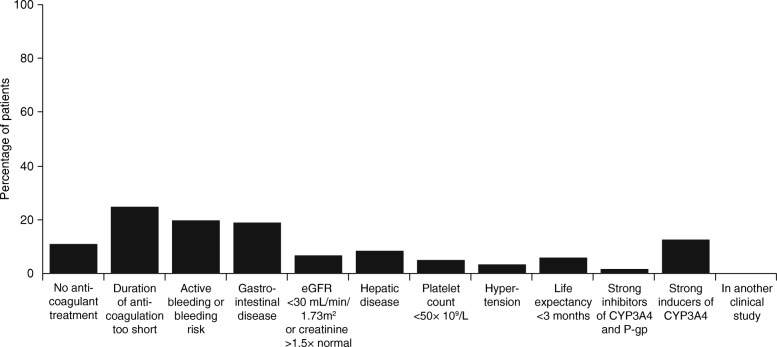
Percentage of the whole population not meeting individual eligibility criteria of the draft EINSTEIN-Jr. protocol. The bars sum to more than 100% because many children would have been excluded by multiple criteria

The main reasons for ineligibility for children aged < 0.5 years were a gestational age < 37 weeks (35.4%), a bodyweight < 2600 g (25.4%), and inadequate oral, nasogastric, or gastric feeding prior to or on the day of their diagnosis of VTE (36.5%). Figure 2 shows the percentage of children not meeting the remaining individual inclusion and exclusion criteria of the draft EINSTEIN-Jr protocol criteria. The main reasons for ineligibility for the entire cohort of children younger than 2 years, were no anticoagulant therapy given (11%), duration of anticoagulant therapy was too short (25%), presence of active bleeding or high bleeding risk (20%), gastrointestinal disease potentially associated with poor absorption (19%), and use of potent CYP 3A4 inducers (13%).

### Estimated cumulative loss of participation in EINSTEIN-Jr

**Fig. 3:**
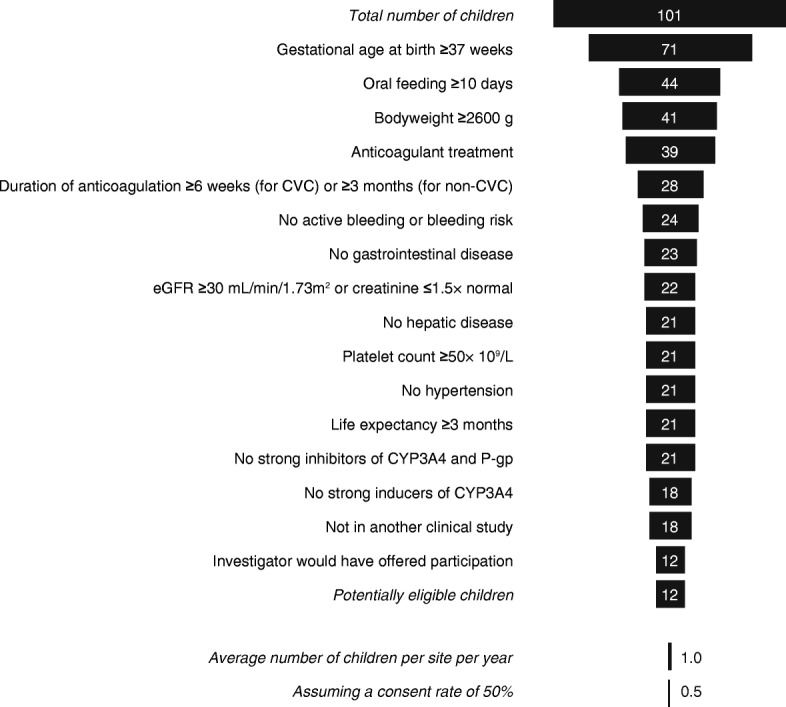
Funnel showing cumulative loss of children aged < 0.5 year due to failure to meet the proposed eligibility criteria. Missing data does not exclude the individual

**Fig. 4:**
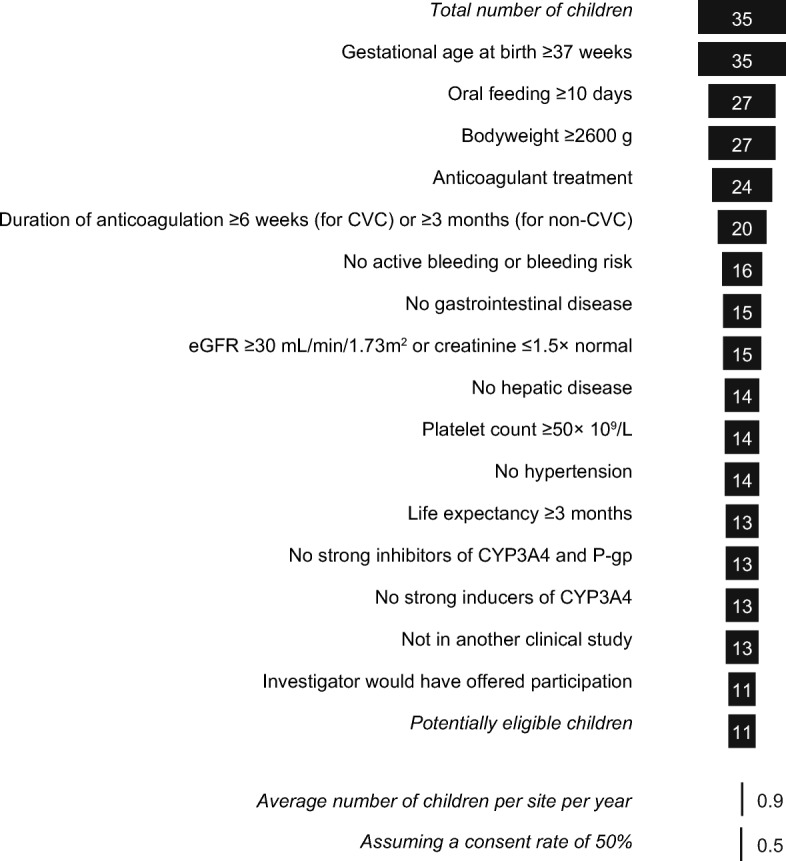
Funnel showing cumulative loss of children aged 0.5–2 years due to failure to meet the proposed eligibility criteria. Missing data does not exclude the individual

For the year 2016, a total of 101 children (aged < 0.5 years) and 35 children (aged 0.5–2 years) were evaluated using the EINSTEIN-Jr eligibility criteria (Figs. 3 and 4). Of these, 18 (17.8%) and 13 (37.1%) would have passed the screen of eligibility criteria of EINSTEIN-Jr., of whom 12 (11.9%) and 11 (31.4%) would have been considered for participation by the clinicians, respectively. This translates into an average number of potentially eligible children per year per site of 1.0 and 0.9, respectively. If a consent rate of 50% is assumed, these numbers will be 0.5 and 0.5 children per year per site, respectively.

## Discussion

This study demonstrated that two-thirds of children with VTE younger than 2 years developed their VTE as a complication of the use of central catheters, with the highest incidence of VTE in the first month of life. In contradistinction to what might be expected given the current pediatric anticoagulant guidelines [1], this snapshot of contemporary clinical practice revealed that no anticoagulant therapy was given to one-tenth of children, whereas one-fourth received a short duration of anticoagulant therapy, respectively. Recurrent VTE rates without anticoagulation, during anticoagulation or shortly after its discontinuation seem comparable to those observed in adults [27–30].

The reasons behind non-adherence to the international guidelines for many children in this analysis was beyond the scope of this study, however, it is likely that it is related to the absence of any randomized, quasi-randomized, or cluster-randomized controlled trials in neonates or young children with a diagnosis of thrombosis [31]. *In addition*, we postulate that it could be related to the presence of serious illnesses contraindicating the use of anticoagulants, apprehension of bleeding risk in the neonatal period, the presentation with minimal clots, and the practice of repeat ultrasound imaging to guide duration of treatment, with continuation of anticoagulation only if recanalization has failed. Whether resolution on ultrasound represents true cure, and abolition of risk of recurrence for catheter or non-catheter related VTE remains to be determined.

The most important reasons which could render children younger than 2 years ineligible for the EINSTEIN-Jr study were a gestational age of less than 37 weeks at birth, a bodyweight less than 2600 g and the requirement for at least 10 days of oral feeding. As a result, an inclusion rate of approximately 0.5 young child per year per site can be anticipated in EINSTEN-Jr. Use of short and flexible treatment durations carries potential to increase recruitment rates and appears to be in keeping with current practice for anticoagulant therapy duration, but raises the spectre of elevating expected recurrence rates.

Based on this analysis, we did not modify the screen of eligibility criteria of young children; however, we decided that young children with CVC-VTE could be treated for a minimum of 4 weeks. Although a treatment duration of less than 3 months carried the potential for increased recruitment in children younger than 2 years who had non-CVC-VTE, we decided to keep the required treatment durations at least 3 months since it is well documented that shorter periods of anticoagulation are associated with increased rates of recurrent VTE in adults [12–14]. We believe that these treatment durations will allow physicians to consider most young children with clinically significant VTE for our study. If physicians consider prolongation of anticoagulation beyond these required durations, the protocol will allow continuation of treatment for CVC-VTE in 1-month increments (up to a total duration of 3 months), and continuation of treatment for other VTE in 3-months increments (up to a total duration of 12 months). Also, repeated imaging will be done at the end of the 1-month treatment period in children with CVC-VTE and at the end of the 3-months treatment period in children with other VTE.

### Potential limitations

Due to the retrospective nature of the study, we believe that our analysis may overestimate the eligibility for EINSTEIN-Jr. of young children with VTE. Although a large consecutive series of children diagnosed with VTE was identified, the physician’s assessment of eligibility may have overlooked conditions and circumstances which at the time of the acute phase could have rendered the child ineligible. Furthermore, our search strategy to identify children with VTE may have been biased towards those who had received anticoagulant therapy, thereby underestimating the number of young children who did not receive anticoagulant therapy. Also, we did not capture whether anticoagulation was given in therapeutic dosages and it is, therefore, possible that some of the considered children received anticoagulation in sub-therapeutic dosages.

## Conclusions

Neonates and young children with thrombosis often have CVC-VTE, and often receive no or only short durations of anticoagulant therapy. Recurrent VTE rates without anticoagulation, during anticoagulation or shortly after its discontinuation seem comparable to those observed in adults. Longitudinal studies to better understand the natural history of VTE in young children are urgently required. Once the population that definitely requires treatment is identified, then randomized clinical trials evaluating anticoagulant strategies are urgently needed to address the question of dose and duration, and to evaluate its benefit/risk in the treatment of VTE in young children. However, recruitment will be very challenging. Allowing short and flexible treatment durations into the EINSTEIN-Jr. phase III study could potentially increase recruitment and permit a representative population of current practice.

## Abbreviations

CVC-VTE: Central venous catheter-related thrombosis
VTE: Venous thromboembolism
